# Tackling racial microaggressions in dental training: a novel approach using comics

**DOI:** 10.1038/s41415-025-8387-y

**Published:** 2025-06-27

**Authors:** Kitty Guo, Christopher Murray, Mike Bartle, Golnar Nabizadeh, Mohammad Islam, Clio Ding

**Affiliations:** 586152392638388080542 School of Dentistry, University of Dundee, United Kingdom; 182719107201756500855 School of Humanities, Social Sciences and Law, University of Dundee, United Kingdom; 800837941996763104940 College of Arts and Social Science, Australian National University, Australia; 151046076256411718971 Art Educator and Comic Artist, Cyborgs and Dinosaurs, Singapore

## Abstract

Microaggressions happen frequently, are under-reported and are often unaddressed. Racial microaggressions are common, and by their subtle and often unintentional nature, can be harder to identify and manage than overt racism. This report will detail the changes being made in a teaching hospital to address racial microaggressions by the creation of a bespoke comic illustrating adapted lived experiences of racial microaggressions.

An online questionnaire was sent to staff and students at Dundee Dental Hospital and Research School collecting examples of racial microaggressions experienced on dental clinics. A collaborative approach with the Scottish Centre for Comics Studies adapted examples of racist encounters obtained from the questionnaire into four distinct comic strips, illustrating common examples of microaggressions experienced on clinics. The comic was published and has been disseminated and discussed at a number of events and has been developed for use in a training workshop.

The comic highlights the emotional response of the recipients, as well as the microaggressive behaviours themselves. The dissemination of the comic has initiated open discussions about racism and has accelerated the development of our anti-racist action plan.

The resulting comic aims to improve visibility and awareness of the manifestations of racism on clinics and has led to the implementation of an interactive workshop on addressing microaggressions. We hope these stories will lead to a greater understanding of how microaggressions can detrimentally impact people. Identifying racial microaggressions allows us to consider our own behaviour and that of others to help create a community that is fair and inclusive.

**Supplementary Information:**

Zusatzmaterial online: Zu diesem Beitrag sind unter 10.1038/s41415-025-8387-y für autorisierte Leser zusätzliche Dateien abrufbar.

## Key points

- The reader will be aware of common themes of racial microaggressions.
- The reader will gain an understanding of the value of comics in information dissemination.
- The reader will gain an understanding of the implications of microaggressions on individuals.

**Supplementary Information:**

Zusatzmaterial online: Zu diesem Beitrag sind unter 10.1038/s41415-025-8387-y für autorisierte Leser zusätzliche Dateien abrufbar.

## Introduction

The prevalence of racism within dentistry and the NHS (National Health Service) is clear, with six out of ten ethnically diverse dentists reporting some form of racial discrimination.^1^ These issues have also been highlighted more generally in higher education following a series of revealing race reports from higher education institutions,^2^^,^^3^^,^^4^ and the new *Safe practitioner framework* of the General Dental Council (GDC) has recommended changes in the recognition and management of racism in dentistry, specifying essential behavioural outcomes related to equality, diversity and inclusion for the Bachelor of Dental Surgery curriculum in the United Kingdom (UK).^5^

Racism is multifaceted, with the most ‘everyday' expression often taking shape as microaggressions, a term introduced by Harvard psychiatrist Professor Chester Pierce in the 1970s.^6^ Forms, types and examples of racial microaggressions are thoroughly explored by Sue *et al.*,^7^ but in the briefest of terms, microaggressions can be considered in contrast to overt racism by their subtle and often unintentional nature. They can include interrogative questioning, stereotyping and jokes, and can be more difficult to address because of an uncertainty of intent. Microaggressions happen frequently, are under-reported and are often unaddressed.^8^ They can cause significant and cumulative psychological distress for those experiencing microaggressions, including anxiety, depression and reduced self-esteem.^9^^,^^10^^,^^11^ Additionally, explorations of medical and nursing staff experiences of racial microaggressions reported negative effects on learning, performance and wellbeing, as well as feelings of burnout.^12^^,^^13^^,^^14^

Though racial microaggressions are commonly experienced by staff and students,^15^ ambiguity, misrecognition and under-reporting dilutes the impact of any action following the occurrence of a microaggression. There may be several underlying reasons for this. First, some may fear the engenderment of any negative consequences for seeking out any action, including distrust or dissatisfaction in the bodies responsible for addressing reports or concerns.^7^^,^^8^^,^^16^Secondly, the person experiencing a microaggression may consider whether or not the micro-event is as harmful as an overt racist attack. Thirdly, does the organisational structure recognise microaggressions?

Idle *et al.*^17^ highlight the concept of ‘everyday harm' and how recognition of subtle but damaging encounters can help prevent the occurrence of microaggressions. Within clinical undergraduate and postgraduate programmes, educators have a responsibility to address the concerns of learners, including supporting their professional development beyond graduation. This includes demonstrating appropriate support for racist encounters, including recognition of events, open dialogue and appropriate and timely management.

This report will discuss stages of the production of an educational comic book^18^ based on the lived experiences of staff and students and Dundee Dental Hospital and School and subsequent dissemination and development of training resources and further actions that have been taken to address racism. This interdisciplinary work grew out of recent collaborations between the Dental School and the Comics Studies Creative Research Hub (CSCRH) which is part of the Division of Humanities in the School of Humanities, Social Sciences and Law at the University of Dundee. CSCRH has developed a range of public information and educational comics, working with various partners, such as the NHS and various charities.

The aim of this project is to contribute to a more inclusive and resilient staff and student community, by improving the recognition of racism and encouraging people to challenge racist behaviour.

## Stage 1: Questionnaire

The first stage of this project involved the identification of examples of racism in clinical settings from staff and students at Dundee Dental Hospital and Research School (DDHRS). This was achieved through the distribution of an online questionnaire hosted on the platform JISC. The questionnaire was distributed through an email invitation to all staff and students at DDHRS. Respondents were asked to share any examples of microaggressions or overt racism that they have received or observed from patients, students or staff while working on clinics. Participants were informed to redact any identifiable information from their responses. Participants were offered the opportunity for their experiences to be used for developing training materials by asking the question: ‘do you give consent for your example(s) to be illustrated and used as part of a training course?'. Consent was obtained from participants of the questionnaire and only responses from those who consented to their examples being illustrated were used for the comic.

The questionnaire was developed with a focus group of staff and students interested in helping our school develop its inclusive curriculum. Membership included two members of the CSCRH, a comic book artist, a clinical lecturer in restorative dentistry, the dental school's equality, diversity and inclusion lead, a student representative, and an NHS staff representative.

The Dental School worked in collaboration with the CSCRH to create a comic book depicting stories of microaggressions experienced in a clinical setting, adapted from the questionnaire responses.

As the aim of this project was to enhance our curriculum, develop new methodologies and explore new mediums for tacking racism, the internal university ethics committee advised that ethical approval was not required because of the service evaluation design.

The 30 responses from the questionnaire identified instances of racial microaggressions and overt racism experienced in clinical environments. The following section will describe the processes involved in developing the responses into a comic book.

## Stage 2: Comic creation process

Once responses from the questionnaire were gathered, they were grouped into four categories by the members of the above-described focus group, based on the types of impact that they may cause. These were: 1) alienation; 2) objectification; 3) inferiority; and 4) questioning of identity. From there, the team set about creating characters and scenarios that drew on the experiences communicated through the questionnaire. It was decided that none of the stories should be biographical but would feature characters whose experiences were an amalgamation of those reported in the survey. This meant that the stories and characters would be authentic and relatable but not traceable to any individual or circumstance. The next stage was to develop the four scripts and to pass these to the artist, Clio Ding, who first produced sketches and layouts, for which the authors provided detailed feedback. Ding then developed the final artwork, including colour and lettering.

The aim of the comics was to use the sequential visual storytelling capabilities of comics to communicate a message about microaggressions in a way that is different from other forms of healthcare communication, such as pamphlets and posters. A key consideration was to employ the strengths of the medium of comics. Scholarship on educational comics, and healthcare comics in particular, has revealed that the formal aspects of comics, including the combination of words and images in a sequential narrative, makes them particularly effective for these kinds of communication.^19^^,^^20^^,^^21^^,^^22^

One of the primary considerations was the style. The team wanted the details in the comic to be rendered quite realistically and the stories had to be grounded in real experiences while using an appealing style that used the power of comics to draw attention to the emotions and subjective aspects of these experiences. The tone of the stories also required careful attention as the message needed to be hopeful as well as honest about the challenges.

We found the way that comics tell stories to be particularly effective for this theme. Many people who experience a microaggression do not react immediately or explicitly. They often appear not to be uncomfortable or offended, but telling these stories in the form of comics allowed us to reveal, through the careful use of thought balloons and captions, the difference between how they might present their responses to the microaggression and how they actually feel and think about what has occurred. Comics are particularly good at presenting ironic juxtapositions between words and images and we found this strategy to be a powerful tool in representing the effect of microaggressions. In several instances, the character is shown reacting in a certain way but the thought balloons reveal what their desired response may actually be. Likewise, the expressions and gestures of these characters reveals the pain, worry and embarrassment that is caused by the microaggressions. Comics are a useful tool in presenting the performative aspect, helping readers to relate to the scenarios and emotions and imagine how they might feel or react in these circumstances.

**Fig. 1:**
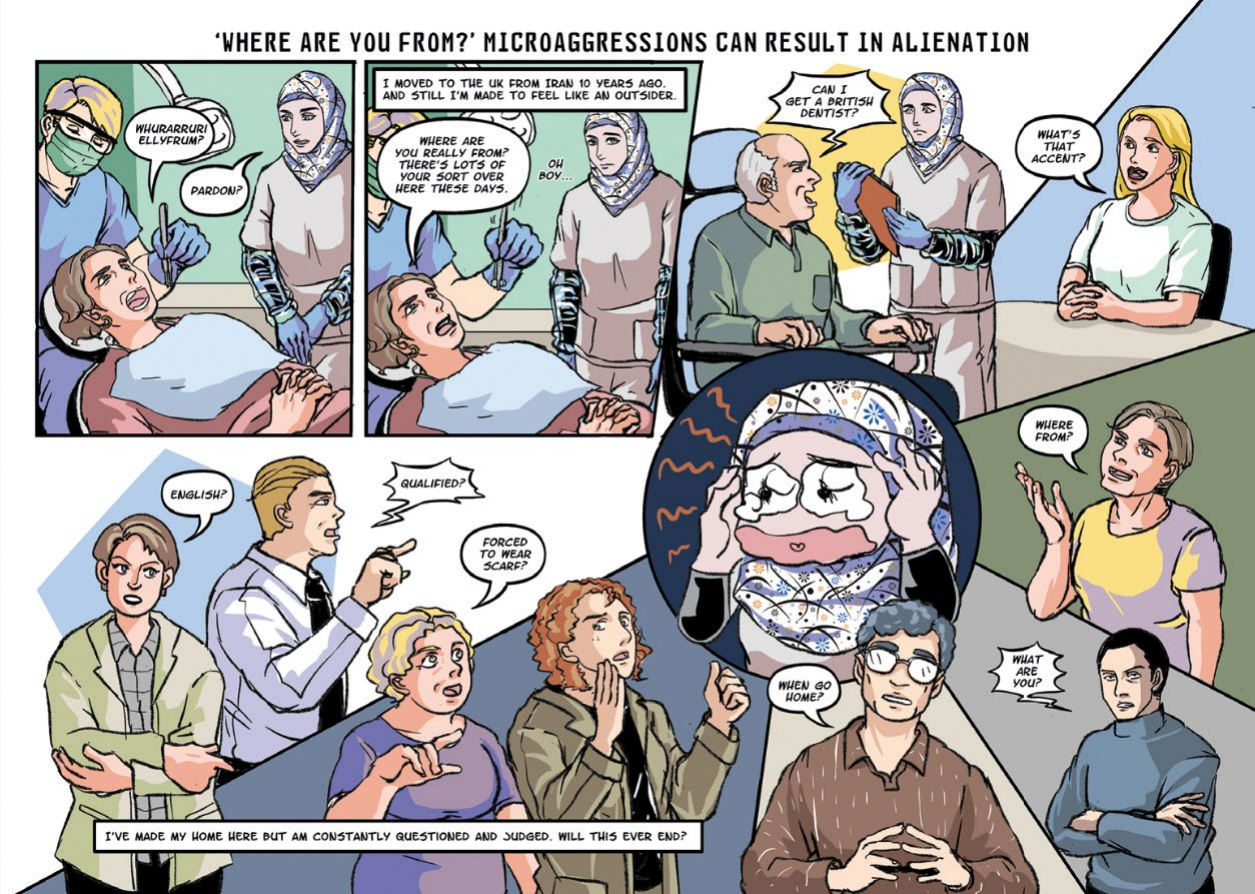
Comic strip depicting alienation

**Fig. 2:**
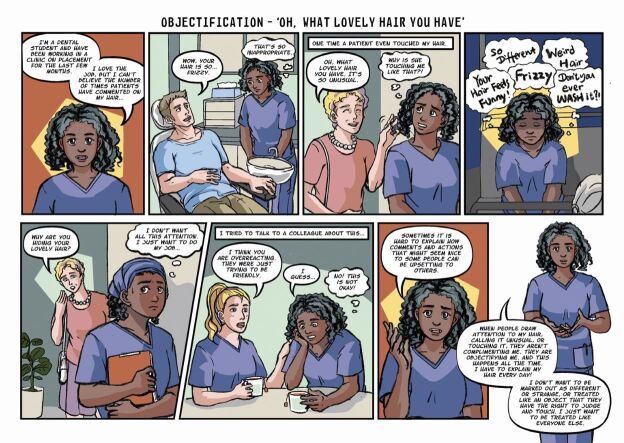
Comic strip depicting objectification

**Fig. 3:**
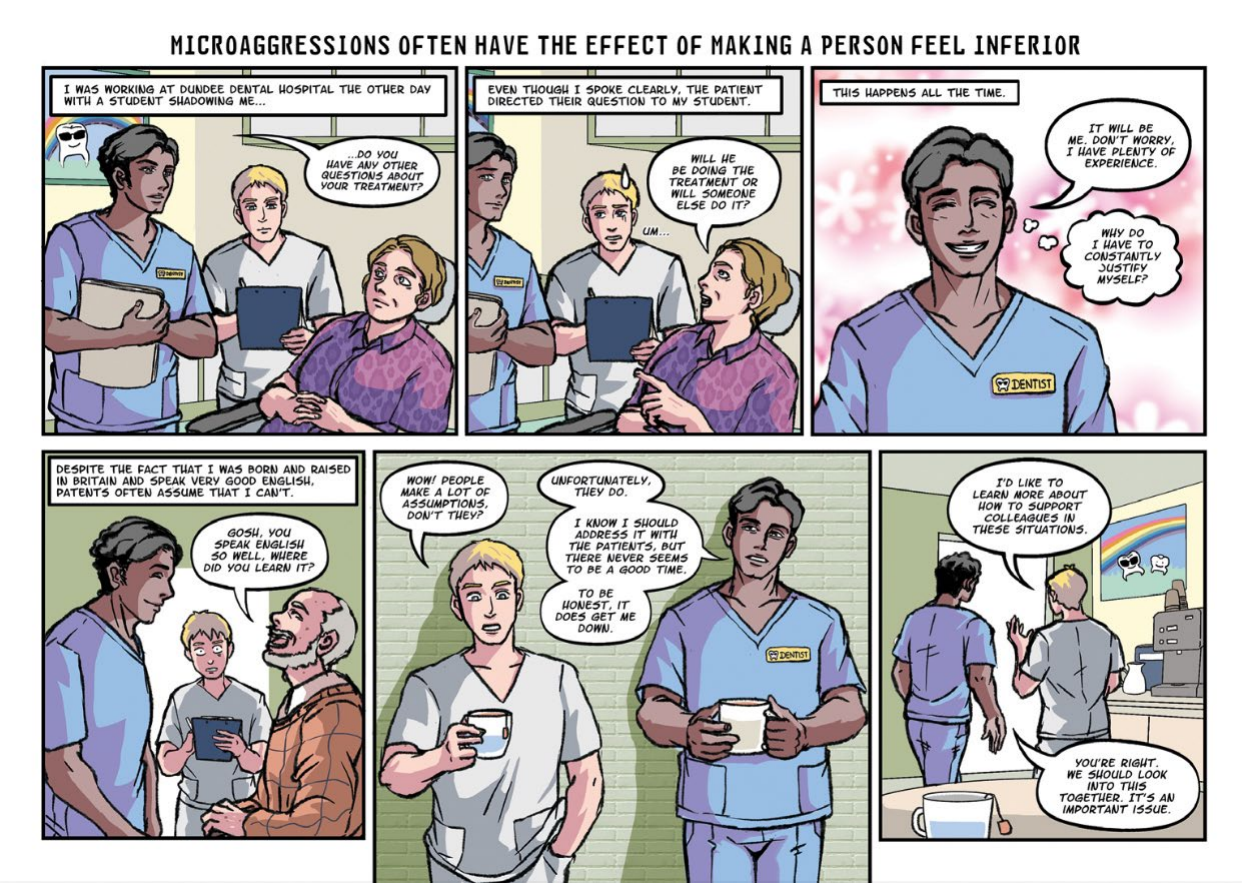
Comic strip depicting inferiority

**Fig. 4:**
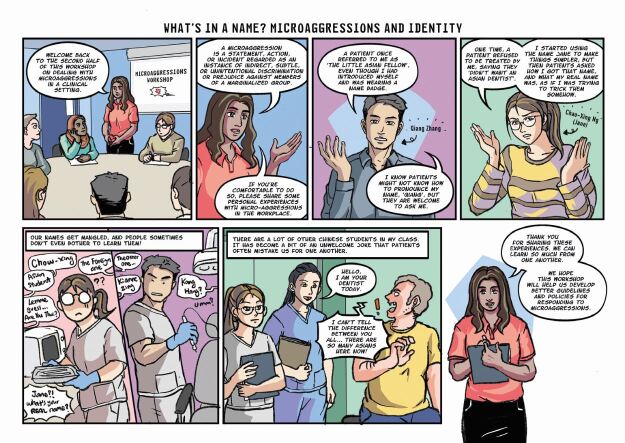
Comic strip depicting issues around identity

The comic strips (Fig. 1, Fig. 2, Fig. 3, Fig. 4) demonstrate not only the act of the microaggressions themselves but use the stylistic and formal properties of comics to illustrate a range of emotional consequences of these experiences.

The four stories depicted a range of commonly occurring racial microaggressions that staff and students experience in dentistry and the NHS. These stories were combined into a comic book. The complete comic is freely available to download and use (see online Supplementary Information).

## Stage 3: Dissemination of the comic book

Following the completion of the comics, it was launched at an event in the University of Dundee during Race Equality Week 2024. Subsequent dissemination included distribution of hard copies to the minority ethnic staff networks in the University and NHS Tayside, as well as to dental students and the Dundee University Student Association. Poster versions of the comic have been installed at various points in the Dental Hospital. The comic has also been shared with the other University of Dundee Protected Characteristics Networks, with initial plans to develop further comics exploring issues experienced by other marginalised groups.

Dialogue generated following the dissemination of this comic has resulted in recommendations that are in progress within Dundee Dental School, outlined in Table 1. The proposed recommendations were reviewed by the School of Dentistry's Equality, Diversity and Inclusion (EDI) Committee and have been integrated into the School's EDI action plan. These were proposed in alignment with the University of Dundee's Race Equality Charter action plan (2022). The plan includes implementation of the comic in workshops for undergraduate and postgraduate students, as well as both NHS and university staff members.

**Table 1:** Recommendations to support staff and students in addressing racism

**Recommendation**	**How it will be achieved**
Have robust reporting mechanisms for racist incidents	People are advised to report racist incidents through the clinical reporting system and to the School's EDI lead
Staff training on microaggressions	Various training workshops will be held throughout the year for staff to educate on how to manage microaggressions
Student training on racism including microaggressions	An anti-racist culture will be embedded into the curricula, in line with the GDC S*afe practitioner framework*, including workshops on what racism is and how to manage microaggressions
Regular minority ethnic support group meetings	The Dental School has started a minority ethnic support group for staff and students to be able to discuss any matters concerning racism in a confidential and supportive group
Utilisation of the microaggressions comic to develop new training resources	The Dental School, in collaboration with NHS Education for Scotland, will incorporate the comic in newly developed anti-racist training resources

## Stage 4: Development of an interactive workshop using the comic

The aim of a workshop using the comic is to use the stories as a starting point for discussions of sensitive topics. Participants can discuss the scenarios presented in the comic and this can be used to stimulate discussion of what could be done differently in these situations, or indeed, as a prompt to encourage participants to share their own experiences or challenge their own assumptions.

The workshop asks the participants to consider the following questions regarding each comic strip:

1. Have you seen this happen?
2. Why might someone find this offensive/uncomfortable?
3. How would you respond if you experienced or observed this behaviour?
4. What would you think if someone told you this happened to them?
5. How would you support a colleague in this situation?

A final prompt asks participants to choose a frame from the comic and respond as if they were the recipient or witness.

This workshop has been delivered to a cohort of postgraduate students at DDHRS and is planned for delivery to the Scottish dental core trainees.

## Discussion

The geographic location of certain dental schools can result in wide discrepancies between the ethnic diversity of the local populations compared with the student populations. For example, 12.9% of people in Scotland have a minority ethnic background,^23^ in stark contrast to the demographics of students in the UK studying medicine and dentistry, where the percentage of students identified as ‘Black, Asian, multiracial or other' was 44.6% in 2023/2024, an increase from 30.1% in 2014/2015.^24^ This difference arises through competitive recruitment of students from the whole of the UK and increasing international recruitment and partnerships, with the total of non-European Union student enrolments in higher education in Scotland, and in the UK overall, doubling in number from 2018 to 2023.^24^ While there is significant ethnic diversity within dentistry, it is important to note that Black groups remain underrepresented in dentistry and dental school admissions.^25^^,^^26^The changing demographics of dental students brings an increasing need for improved understanding of racism experienced by marginalised students and staff.

The comic has increased visibility of microaggressions that have been experienced by the staff and students in this institution and has given a voice to those who experience this. This has resulted in an increased confidence to have open dialogue about racism, reflected by the positive engagement of the first Dental School Ethnic Minority Support Group and a request from attendees to maintain regular meetings. It has also been recognised by the wider University of Dundee community where the authors have been awarded with a Dundee Difference Award in the inclusivity champions category. It is hoped that this acknowledgement of common examples may increase reporting of racist incidents by overcoming reporting barriers, including the fear that an incident won't be taken seriously, or any uncertainty that what an individual has experienced justifies formal reporting.

The use of a comic has been effective at revealing aspects of a microaggressive communication that is not always immediately obvious to an observer. This is particularly useful when illustrating microaggressions, which by nature are subtle and often not responded to by the recipient. The benefit of using the medium of comics is in its ability to portray the emotions or thoughts of the recipient which may not be readily expressed for various reasons. It has helped greatly in our aims to illustrate how microaggressions can affect the recipient.

The themes identified during the production of this comic align with previously described experiences of racism among minority ethnic medical students in the UK^13^^,^^27^and the wider population.^14^^,^^28^^,^^29^Though the comics are set in the context of a dental school, the scenarios have clear relevance across other subject areas and departments, particularly those with public-facing roles, including medicine, nursing and education. The comic can be used directly by other institutions as a training resource, including for use in anti-racist workshops, applicable to the new GDC *Safe practitioner framework* learning outcomes and behaviours listed in Table 2.

**Table 2:** Learning outcomes and behaviours taken from the GDC's *Safe practitioner framework*

**Behaviours**	
P (B)1	Treat your patients, members of the public and your colleague with dignity and respect and without discrimination
P (B)3	Demonstrate cultural competence, accepting and respecting the diversity of patients and colleagues
P (B)5	Speak up to protect others from harm
P (B)6	Raise concerns where appropriate about your own or others' health, behaviour or professional performance
P (B)13	Proactively address discriminatory language, behaviour and microaggressions from colleagues, patients and other professionals
P (B)15	Work in partnership with colleagues to develop and maintain an effective and supportive environment which promotes safety and wellbeing of the patient and dental team
S (B)2	Recognise personal assumptions, biases and prejudices and manage the impact of these on patient care and professional behaviour with colleagues, patients and wider society
**Learning outcomes**	
P 1.3	Describe diversity, equality, inclusion and discrimination and the underpinning legislation, and explain how to apply these principles to manage patients with protected characteristics and work within the dental team
P 1.4	Explain the cultural competence and its relevance in assessing the needs and planning care for patients from diverse backgrounds

## Conclusion

The open discussion generated since the launch of the comic has accelerated the identification and implementation of an anti-racist training workshop in the School of Dentistry, focusing on microaggressions that arise in a clinical environment. Additionally, a support group has been founded, following in the footsteps of the School of Health Sciences at the University of Dundee, welcoming open discussion of any concerns regarding racism for staff and students, as well as clarification and dissemination of the appropriate reporting procedures to be followed. The development of this well-received comic can be translated to other areas of uncertain or overlooked behaviours with negative effects, including LGBT+ and disability issues, which is currently being explored in the University of Dundee, as well as other environments outwith the dental clinic. We welcome any comments and feedback to the main author.

## Supplementary Information

Experiencing Microaggressions Comic (PDF 18MB)

## Data Availability

Due to the nature of the data collected, supporting data are not available.
