# Supplementary material for: Tackling racial microaggressions in dental training: a novel approach using comics
Source: Br Dent J. 2025 Jun 27;238(12):943–8. doi: 10.1038/s41415-025-8387-y (PMC12204854; doi:10.1038/s41415-025-8387-y)
Supplement: Supplementary file 1 — Experiencing Microaggressions Comic (PDF 18MB) [file 41415_2025_8387_MOESM1_ESM.pdf]

EXPERIENCING MICROAGGRESSIONS

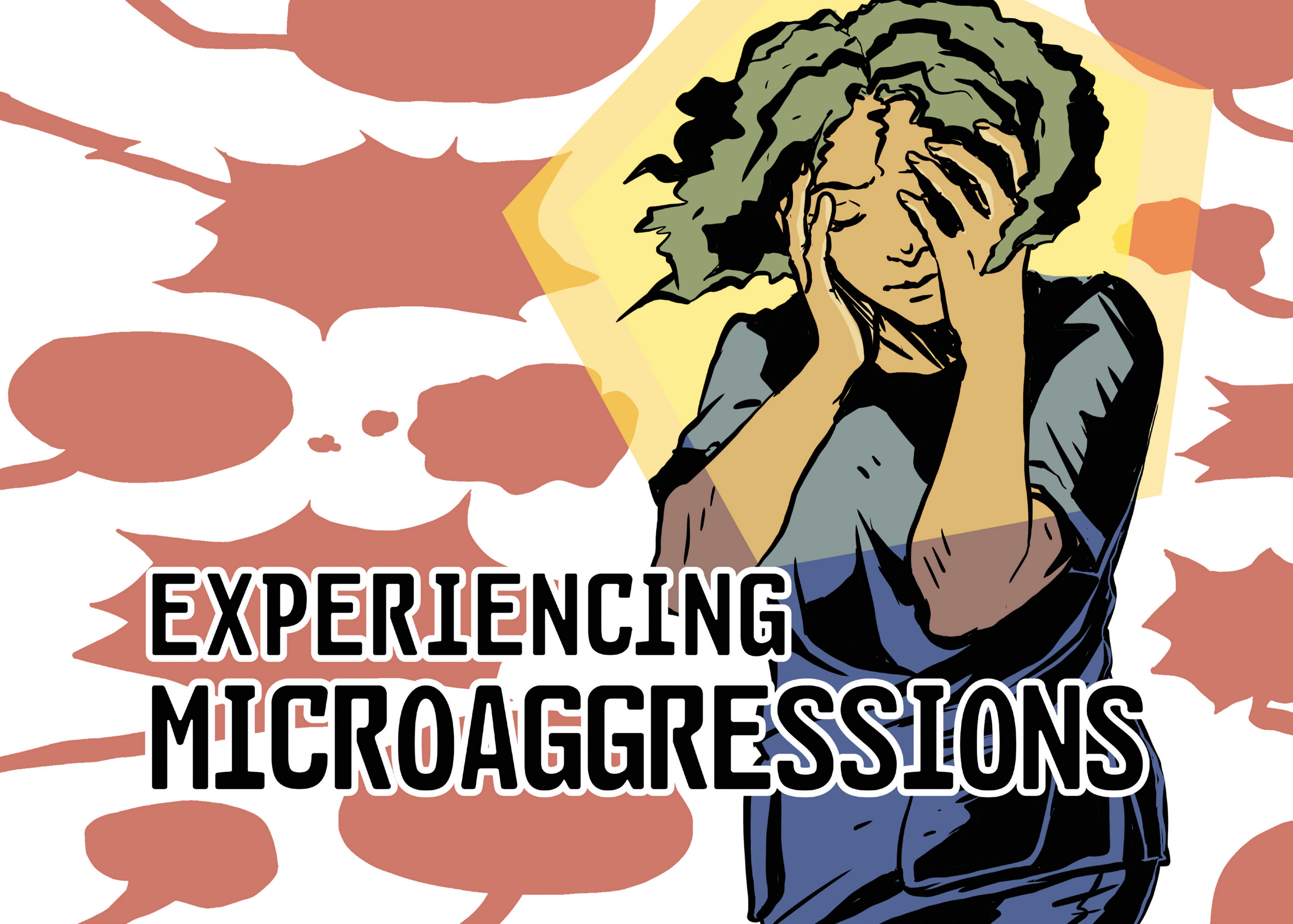

EXPERIENCING MICROAGGRESSIONS

A microaggression is ‘a statement, action, or incident regarded as an instance of indirect, subtle, or unintentional discrimination or prejudice against members of a marginalized group such as a racial minority’ (OED). Sometimes it’s hard to respond to microaggressions because individuals may feel uncertain whether a microaggression has occurred, leaving them unsure about how to respond or if doing so will help. Responding directly to a microaggression may bring about negative consequences, including accusations of being oversensitive or paranoid, or being told that such responses confirm stereotypes about minorities. This can result in these real experiences becoming invalidated, which is harmful for all concerned.

This comic is designed to start conversations about microaggression by presenting four stories from staff and students from our community based on their experiences of racist microaggressions. We hope these stories will lead to more conversations about microaggressions generally, and racist microaggressions, leading to a greater understanding of how microaggressions can detrimentally impact people. Identifying racial microaggressions allows us to consider our own behaviour and that of others to help create a community that is fair and inclusive.

The examples illustrated in these stories have been adapted from real stories provided by staff and students at Dundee Dental Hospital and Research School. These are an amalgamation of experiences and are not a direct representation of any individual example. Consent was obtained for use of these stories.

This comic was written by Chris Murray, Golnar Nabizadeh, Kitty Guo, and Mohammad Islam. The artwork was created by Clio Ding, with production by Damon Herd.

This project was funded by The Quality Assurance Agency for Higher Education.

Experiencing Microaggressions, August 2023, published by UniVerse, University of Dundee, Nethergate, Dundee, Scotland, UK. All Rights Reserved. Production by Damon Herd.
All work created for this comic is © University of Dundee and the creators.

'WHERE ARE YOU FROM?' MICROAGGRESSIONS CAN RESULT IN ALIENATION

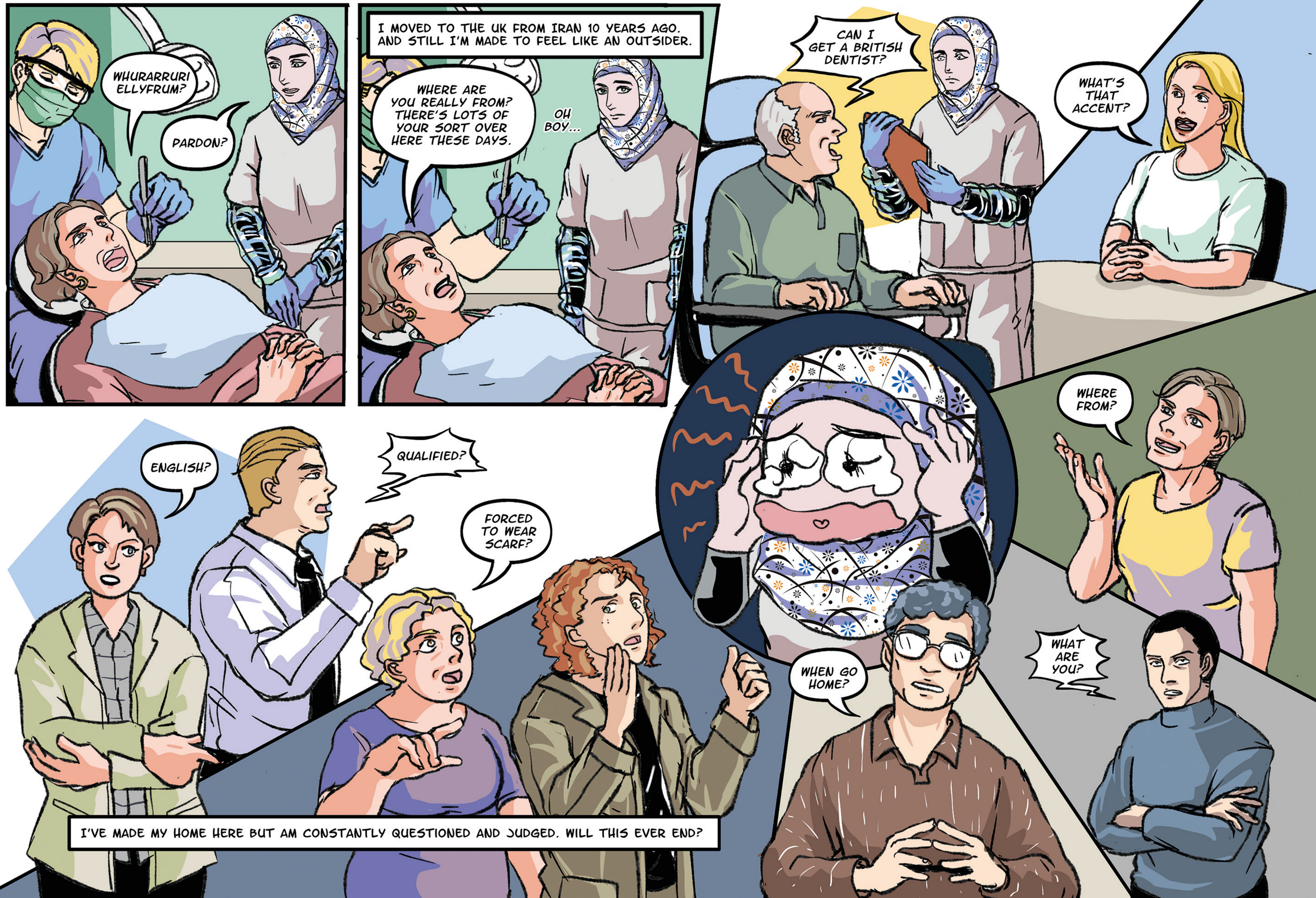

OBJECTIFICATION – 'OH, WHAT LOVELY HAIR YOU HAVE'

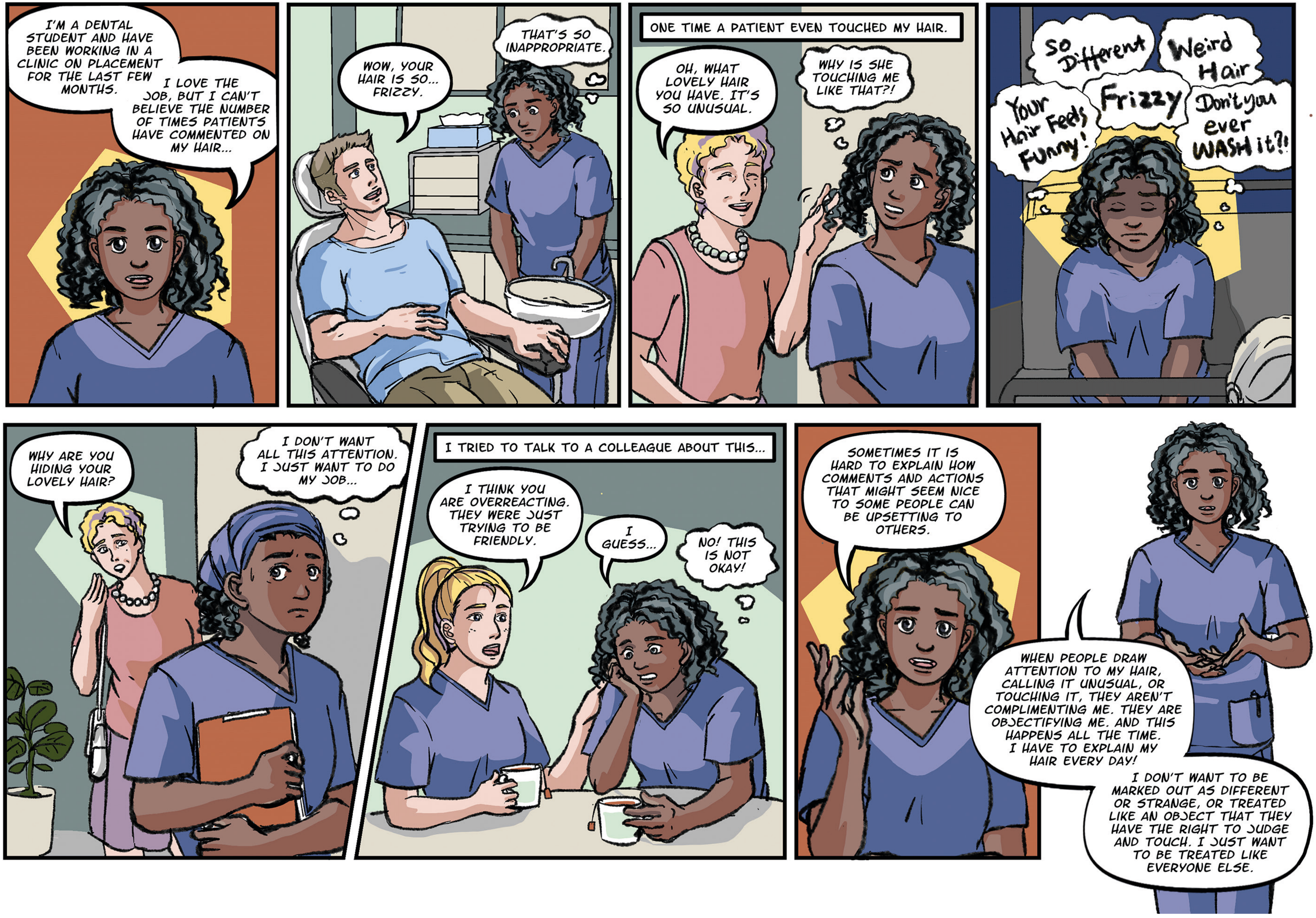

MICROAGGRESSIONS OFTEN HAVE THE EFFECT OF MAKING A PERSON FEEL INFERIOR

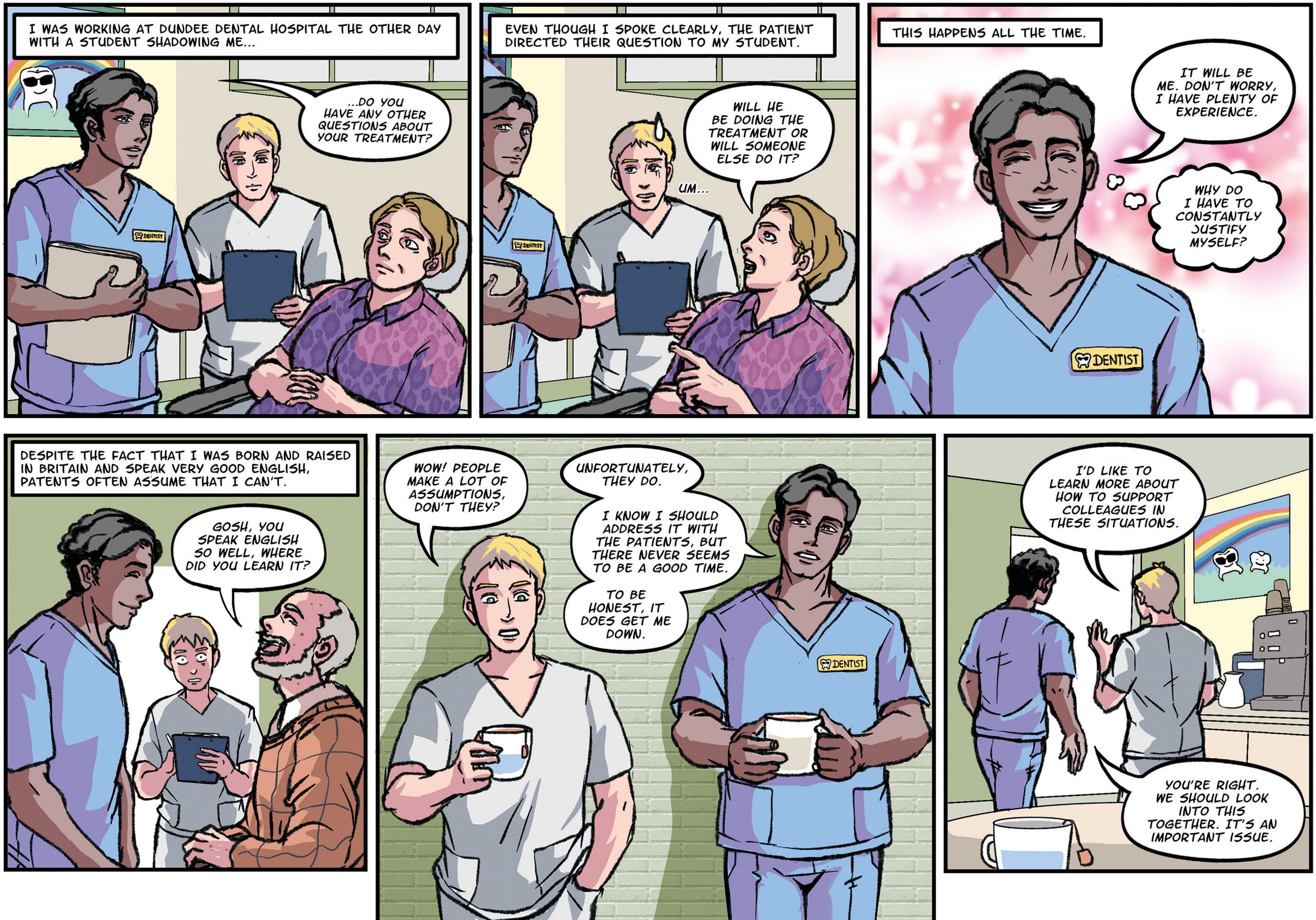

WHAT'S IN A NAME? MICROAGGRESSIONS AND IDENTITY

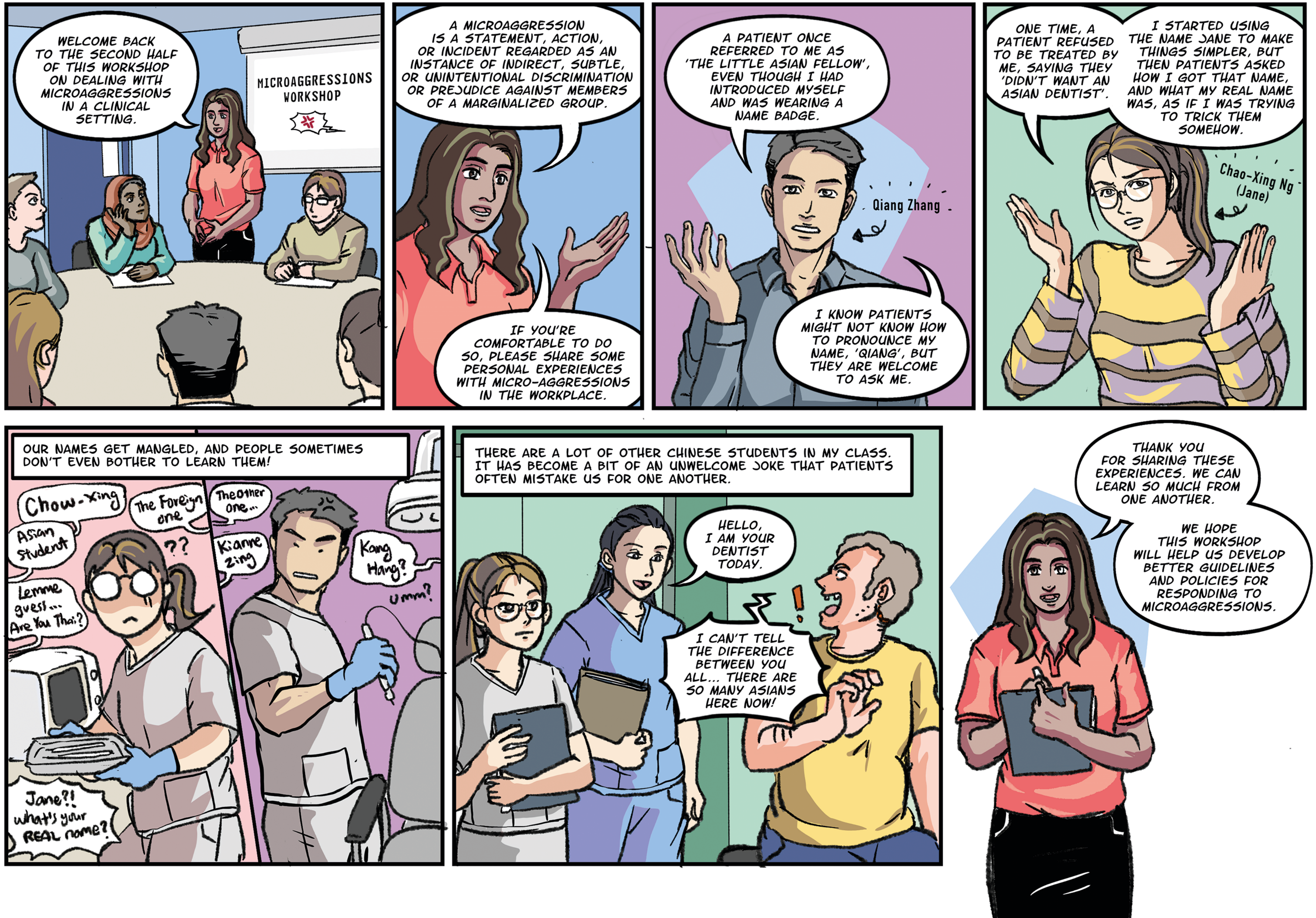

CONTRIBUTOR BIOS

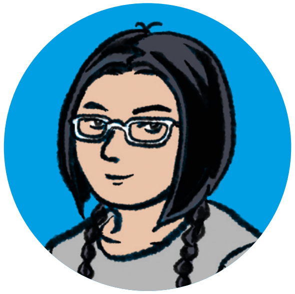

Clio Ding (@clio.ding) is a graduate of the Comics & Graphic Novel MLitt course from University of Dundee. Based in Singapore, Clio teaches art, draws and writes about comics when not being preoccupied with snacks and goldfish.

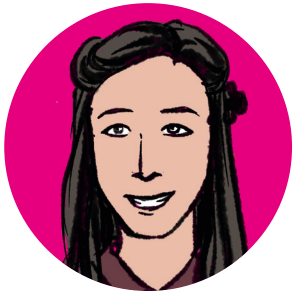

Kitty Guo is a Clinical Lecturer in the School of Dentistry and obtained funding from the Quality Assurance Agency for Higher Education to support this project.

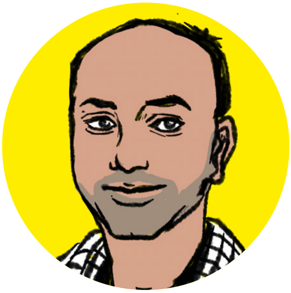

Mohammad Islam is a Lecturer in Preclinical Sciences and the Equality, Diversity and Inclusion lead for the School of Dentistry, University of Dundee. He loves travelling and gardening.

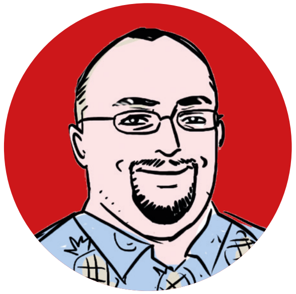

Chris Murray is Professor of Comics Studies at the School of Humanities, Social Sciences and Law at the University of Dundee. He is the director of the Scottish Centre for Comics Studies and has written and edited several comics for research and public engagement purposes.

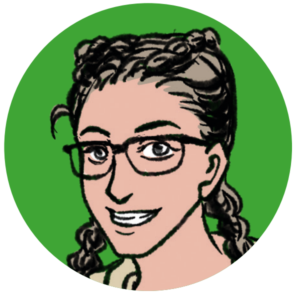

Golnar Nabizadeh is Senior Lecturer in English and Equality, Diversity and Inclusion Lead for the School of Humanities, Social Sciences and Law at the University of Dundee.

With thanks to all those who participated in the microaggressions survey.

A microaggression is ‘a statement, action, or incident regarded as an instance of indirect, subtle, or unintentional discrimination or prejudice against members of a marginalized group such as a racial minority’ (Oxford English Dictionary). It is important to realise that while a microaggression is, by definition, a ‘small’ transgression, perhaps even one that might go unnoticed by many, their impact may not be small. Microaggressions can have devastating consequences. This comic features stories that demonstrate the nature and effect of microaggressions in a clinical setting, drawing on the experiences of staff and students in the School of Dentistry.

University of Dundee

UniVerse

QAA Scotland
